# A novel group of avian astroviruses from Neotropical passerine birds broaden the diversity and host range of *Astroviridae*

**DOI:** 10.1038/s41598-019-45889-3

**Published:** 2019-07-02

**Authors:** Izaskun Fernández-Correa, Daniel A. Truchado, Esperanza Gomez-Lucia, Ana Doménech, Javier Pérez-Tris, Jonas Schmidt-Chanasit, Daniel Cadar, Laura Benítez

**Affiliations:** 1 Department of Genetics, Physiology and Microbiology, Faculty of Biology, Complutense University of Madrid, José Antonio Novais, 12, 28040 Madrid, Spain; 2 Department of Biodiversity, Ecology and Evolution, Faculty of Biology, Complutense University of Madrid, José Antonio Novais, 12, 28040 Madrid, Spain; 3 Department of Animal Health, Faculty of Veterinary Medicine, Complutense University of Madrid, Avda. Puerta de Hierro s/n, 28040 Madrid, Spain; 4 Bernhard-Nocht-Institut fur Tropenmedizin, WHO Collaborating Centre for Arbovirus and Haemorrhagic Fever Reference and Research, National Reference Centre for Tropical Infectious Diseases, Bernhard-Nocht-Strasse 74, 20359 Hamburg, Germany; 5 University of Hamburg, Faculty of Mathematics, Informatics and Natural Sciences, Hamburg, Germany

**Keywords:** Viral evolution, Metagenomics

## Abstract

Metagenomics is helping to expand the known diversity of viruses, especially of those with poorly studied hosts in remote areas. The Neotropical region harbors a considerable diversity of avian species that may play a role as both host and short-distance vectors of unknown viruses. Viral metagenomics of cloacal swabs from 50 Neotropical birds collected in French Guiana revealed the presence of four complete astrovirus genomes. They constitute an early diverging novel monophyletic clade within the *Avastrovirus* phylogeny, representing a putative new astrovirus species (provisionally designated as *Avastrovirus 5*) according to the International Committee on Taxonomy of Viruses (ICTV) classification criteria. Their genomic organization shares some characteristics with *Avastrovirus* but also with *Mamastrovirus*. The pan-astrovirus RT-PCR analysis of the cloacal samples of 406 wild Neotropical birds showed a community-level prevalence of 4.9% (5.1% in passerines, the highest described so far in this order of birds). By screening birds of a remote region, we expanded the known host range of astroviruses to the avian families Cardinalidae, Conopophagidae, Furnariidae, Thamnophilidae, Turdidae and Tyrannidae. Our results provide important first insights into the unexplored viral communities, the ecology, epidemiology and features of host-pathogen interactions that shape the evolution of avastroviruses in a remote Neotropical rainforest.

## Introduction

The last decades have witnessed a broadening of the global diversity of viruses thanks to rapidly evolving random amplification sequencing technology. Next Generation Sequencing (NGS) has revolutionized our knowledge by not only uncovering the diversity of viruses at an unprecedented rate, but also by allowing to gain insight into their ecology and distribution, helping to disentangle the network that connects viruses with their hosts and the geographical scenario in which such interactions take place^1^. However, deciphering the global virome and its structure becomes challenging not just because of the technical difficulties associated with the screening of diverse viruses, but because their many hosts are often understudied, particularly those from remote areas. The Neotropical region is one such example; being among the most biodiverse realms on Earth, Neotropical rainforests are candidates to host and conserve relevant parts of the virosphere diversity^2^. However, the knowledge of Neotropical viral diversity and their hosts is in its infancy. Given that zoonotic viruses are considered the most probable causative agents of emerging diseases^3^ and that mammal and avian species are their main hosts^4,5^, the study of the wildlife viral diversity and their ecological context in these remote areas will provide important information for public and animal health to prevent potential new emerging viral epidemics^2,6^. For example, the great diversity of the Neotropical fauna harbors the risk of emergence of new infections in domestic animals and humans that might be in contact with these birds or ecosystem because of human impact on nature. Conversely, the valuable biodiversity of these regions may be threatened by invasive viruses imported into the Neotropics^7,8^. There is growing evidence that the risk of pathogen transfer is increasing in both directions^9–11^, and a comprehensive characterization of the virome of the local fauna may be instrumental in preventing any harmful impact. However, this important knowledge would hardly become available without exploratory approaches, which address the discovery and describe the diversity of viruses that affect non-model host species.

In the last decade, astroviruses have been one of the virus families in which the number of their potential hosts has quadrupled thanks to the use of metagenomic studies^12,13^. New astroviruses have been discovered as part of the intestinal virome of wildlife, including bats^14^ and especially birds^15^, although information about the presence and diversity of astroviruses in wild birds is far from being complete. Novel astrovirus genotypes have been discovered in cloacal swabs or fecal samples in a variety of bird species^15–17^, although observed prevalence was usually very low.

The family *Astroviridae* comprises two genera, *Mamastrovirus*, which infect mammals, and *Avastrovirus*, which infect birds. Astroviruses are non-enveloped icosahedral viruses, with a diameter of 28–30 nm. The genome of astroviruses consists of a positive sense linear single-stranded RNA molecule between 6.4 and 7.9 kb with three open reading frames (ORFs): ORF1a, ORF1b and ORF2. ORF1a and ORF1b encode two non-structural polyproteins (nsp1a and nsp1ab) which include a protease and a RNA-dependent RNA-polymerase (RdRp). In the overlapping region between ORF1a and ORF1b there is a distinctive frameshifting mechanism consisting of a heptanucleotide and a stem-loop structure^18^. ORF2 encodes a polyprotein, which is the precursor of viral capsid proteins. This polyprotein is translated from a viral subgenomic RNA (sgRNA) and has two main domains: a highly conserved N terminal and a hypervariable C terminal^19^. A viral genome-linked protein (VPg) with a key role in genome replication or protein synthesis is also encoded in the genome^20,21^. This protein is linked to the 5′-end of astrovirus genome and it possesses a TEEEY-like residue that has been demonstrated to be related to viral infectivity in human astroviruses^20^.

In domestic birds, enteritis is the most frequent clinical sign associated with astrovirus infections, although they have also been correlated with other pathological conditions such as nephritis or hepatitis^22,23^. However, astrovirus infections usually occur asymptomatically in this group of animals, only causing mild disease. The most frequent transmission route is fecal-oral^19,24^, a circumstance that increases the risk of cross-infection among species that share habitat or get in close contact, for instance, in farms. Furthermore, astroviruses are also important for public and environmental health as a causative agent of encephalitis in cattle^25^.

The classification of astroviruses has been redefined several times since they were first discovered in 1975^13^. According to ICTV, the current classification does not correspond to the phylogeny of this group of viruses, being based on the host and the genetic distances (p-dist) among complete amino acid sequences of the capsid region (ORF2). The average genetic distance between groups of *Avastroviruses* is 0.704, and within the same group it ranges between 0.576 and 0.741^13,24^. The *Astroviridae* study group of ICTV also establishes that viruses with p-dist > 75% identity in the complete protein sequence of ORF2 should be considered as members of the same species^13^. Currently, there are three recognized species of the genus *Avastrovirus*. *Avastrovirus 1* is apparently specific of the Galloanserae, with isolates from turkey (Turkey astrovirus-1; TAstV-1), chicken (Chicken astrovirus; CAstV), duck (Duck astrovirus; DAstV), guineafowl (Guineafowl astrovirus; GFAstV) and goose (Goose astrovirus; GAstV). *Avastrovirus* 2 includes avian nephritis virus 1 and 2 (ANV-1 and ANV-2), and has been found mainly in chickens, but also in turkeys, ducks and pigeons sporadically. *Avastrovirus* 3 includes isolates type 2 and 3 from turkey (TAstV-2 and TAstV-3). Therefore, most of the known data on the diversity of *Avastrovirus* comes from poultry studies, with an evident lack of information from wild species, a circumstance which is usual for most avian diseases^26^.

Using a metagenomic approach aiming to characterize the cloacal virome of birds from the primary rainforest of the Guianan shield (one of the most remote Neotropical regions) we observed a high number of viral reads related to astroviruses. Astrovirus-like contigs were assembled and four complete astrovirus genomes were successfully recovered, which we provisionally named Passerine astrovirus (PasAstV)-1-4. These four newly discovered astroviruses exhibit little similarity both among one another and with other representatives of the *Avastroviru*s genus. Therefore, we set out to describe the genomic features and hosts of these viruses, thereby contributing to broaden the knowledge of the diversity and host range of this virus family. We also placed them in the phylogeny of *Astroviridae*, where they represent a putative novel viral species.

## Results

### Diversity and host range of astroviruses

The screening by pan-astrovirus RT-PCR of all 406 cloacal samples retrieved 20 individuals positive for astrovirus, representing a prevalence of 4.9%. Although our samples included birds of six different orders (Apodiformes, Columbiformes, Coraciiformes, Falconiformes, Passeriformes and Piciformes), all positive individuals were passerines. Most of the infected birds belonged to the family Thamnophilidae, but other families such as Cardinalidae, Conopophagidae, Furnariidae, Tyrannidae and Turdidae were also positive for viral infection (Table 1). Attempts to isolate the novel astroviruses detected here were not possible due to very limited availability of the amount of samples.

**Table 1:** Percentage of birds positive for astrovirus within each of the avian families that provided positive samples in French Guiana.

Family	Positive birds for astrovirus	Captured birds	% infected/sampled birds
Furnariidae	3	70	4
Thamnophilidae	13	170	8
Turdidae	1	8	13
Tyrannidae	1	40	3
Others	2	118	2

**Figure 1:**
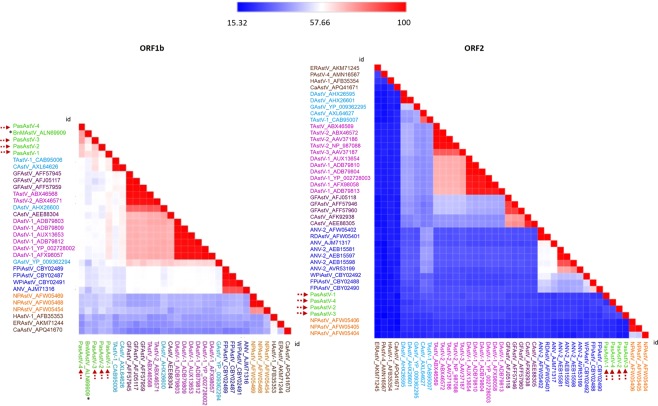
Colour-coded pairwise identity matrix generated from complete ORF1b and ORF2 amino acid sequences of the four novel PasAstV and representative members of the *Astroviridae* family. The PasAstV-1-4 are marked with arrows and the black-naped monarch astrovirus isolate (BnMAstV) is marked with an asterisk. Each coloured cell represents the percentage identity score between two sequences. A colour key indicates the correspondence between pairwise identities and the colours displayed in the matrix. The *Avastrovirus* strains are colored according to the ICTV current species and our proposed new classification (see Fig. 4). Difference in size between the two heatmaps is due to the different availability of sequences for both ORFs in Genbank.

Identity matrices (Fig. 1) showed high divergences of the four novel astroviruses from French Guianan passerine birds, compared to previously known astroviruses. Amino acid sequence identity of capsid protein (ORF2) varied from 35% to 93% within the genus *Avastrovirus*, but we found lower identities when we compared these four novel genomes to one another (24–29%), or when we compared them to other avastroviruses (15–36%). An ORF1b and ORF2 amino acid distance matrix analysis generated with heatmaps confirmed the clear demarcation between passerine avastroviruses and relatives of other *Avastrovirus* genus (Fig. 1). Genetic distance analysis also indicated that the passerine avastroviruses can be further subgrouped into four supported clades: *Passerine astrovirus 1* (PasAstV-1), *Passerine astrovirus 2* (PasAstV-2), *Passerine astrovirus 3* (PasAstV-3) and *Passerine astrovirus 4* (PasAstV-4), since distance values among these four astroviruses double the values found within the other *Avastrovirus* groups (Table 2). The sequences of the complete genomes of the four PasAstV were deposited in GenBank under accession numbers MK096773-76. The closest relative to all four PasAstV in databases was the only one passerine astrovirus described, found in a black-naped monarch. Its genome is partially sequenced and no data related to ORF2 is available, but it shows a 59–63% amino acid identity in ORF1b (RdRp) with the four PasAstV.

**Table 2:** Within genetic distances (p-dist) of the ORF2 amino acid sequence for the phylogenetic groups *Avastrovirus 1*–*5*.

Species	p-dist	Error
*Avastrovirus 1*	0.449	±0.013
*Avastrovirus 2*	0.394	±0.014
*Avastrovirus 3*	0.146	±0.010
*Avastrovirus 4*	0.307	±0.015
*Avastrovirus 5*	0.671	±0.015

### Genomic characterization of the four PasAstV

**Figure 2:**
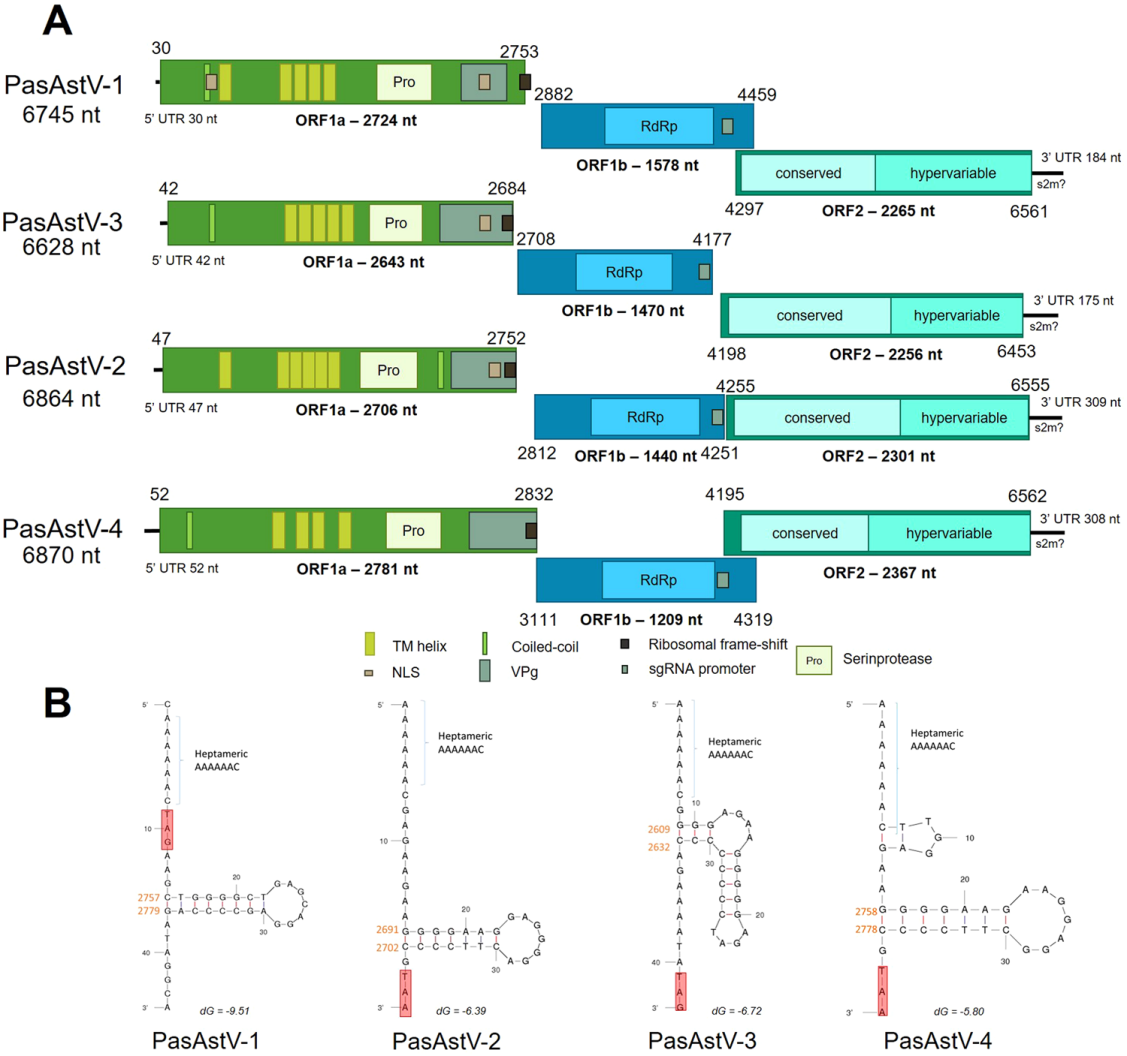
(**A**) Schematic representation of the genomic organization of the four novel PasAstV. NLS, nuclear localization sequence; VPg, viral protein associated with the genome; RdRp, RNA-dependent RNA polymerase; sgRNA, subgenomic RNA; s2m, stem-loop 2-like. (**B**) Prediction of secondary RNA structure of the ribosomal frame-shift at the 3′ end of ORF1a. The termination codon of the ORF is shown in a red rectangle.

**Figure 3:**
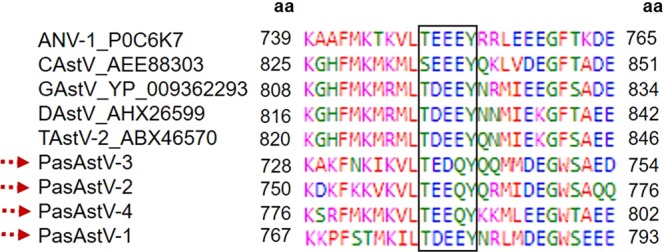
Alignment of the partial protein sequences of the ORF1a of passerine astroviruses (PasAstV-1-4) and some *Avastrovirus* strains at the end of ORF1a (VPg region). The sequence variation is given with respect to the conserved VPg TEEEY motif (black rectangle).

The genomic organization of the four novel PasAstV complete genomes showed that each ORF is in a different reading frame for PasAstV-1 and PasAstV-3. ORFs 1b and 2 of PasAstV-2 are in +1 frame, while PasAstV-4 has ORF1a and ORF2 in the same frame and the ORF1b on +1 frame (Fig. 2A). None of them have exhibited the conserved genomic RNA promoter sequence at 5′ UTR^19^ as based on the Interprosan analyses results using Geneious v11. PasAstV-2, PasAstV-3 and PasAstV-4 preserve the stem-loop structure followed by the ribosomal frame-shift heptameric signal (AAAAAAC) at the 3′ end of ORF1a like the rest of *Avastrovirus*. However, the setting of the ORF1a stop codon in PasAstV-1, between the stem-loop structure and the heptameric signal, shows a typical *Mamastrovirus* structure^19^ (Fig. 2B). Distinctive sgRNA promoter sequences (5′-AUUUGGAGNGGNGGACCNAAN-3′) were found at the 3′ end of the four ORF1b^19^. A structure similar to the stem-loop 2-like (s2m) described for astroviruses^19^ was also observed at the 3′ end of PasAstV-1, PasAstV-2 and PasAstV-3, but not in PasAstV-4. Several conserved protein domains were found in each PasAstV ORF (Table S1) except for a hypervariable region in ORF1. The VPg sequences, with some amino acid variations, were conserved in all four novel astroviruses (Fig. 3).

### Phylogeny and taxonomy

**Figure 4:**
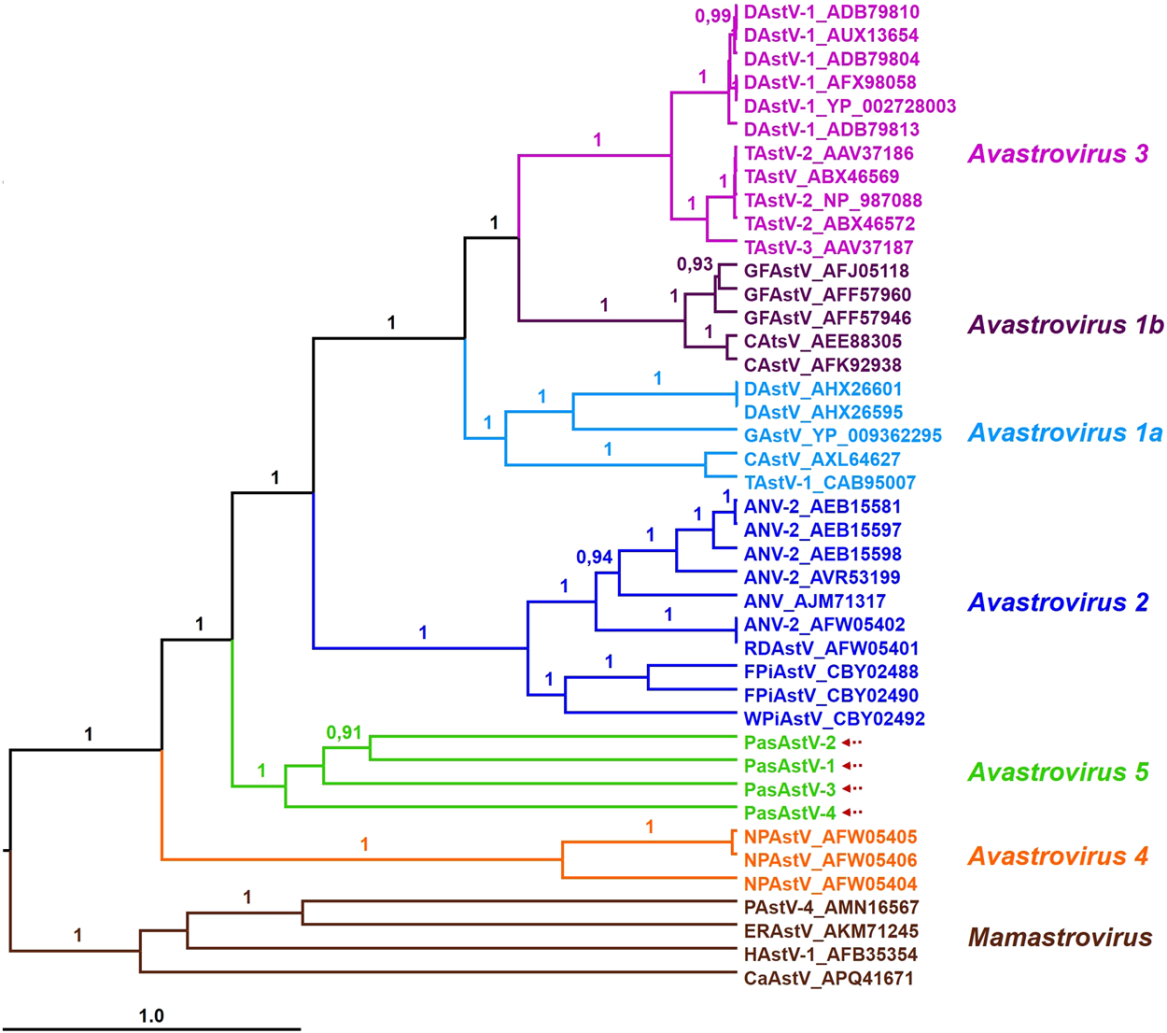
Bayesian maximum clade credibility (MCC) tree based on complete amino acid sequences of ORF1 showing the phylogenetic placement of the four novel PasAstV from this study compared with representative members of the *Avastrovirus* genus. Bayesian posterior probabilities (≥ 90%) are indicated at the nodes. The main lineages are indicated to the right of the tree. Taxon information includes strain names and GenBank accession numbers. The *Avastrovirus* strains are colored according to the ICTV current species and our proposed new classification. Scale bar indicates mean number of amino acid substitutions per site. Four representative members of *Mamastrovirus* are included as outgroup. ANV, Avian nephritis virus; CAstV, Chicken astrovirus; CaAstV, Canine astrovirus; DAstV, Duck astrovirus; ErAstV, European roller astrovirus; FPiAstV, Feral pigeon astrovirus; GAstV, Goose astrovirus; GFAstV, Guinefowl astrovirus; HAstV; Human astrovirus; NPAstV, Northern pintail astrovirus; PAstV, Porcine astrovirus; PasAstV, Passerine astrovirus; RDAstV, Rock dove astrovirus; TAstV, Turkey astrovirus; WPiAstV, Wood pigeon astrovirus.

The analysis carried out to remove potential ORF2 recombinants from our dataset showed no recombination events in the sequences of the four PasAstV. The Bayesian maximum clade credibility (MCC) tree of astrovirus ORF2 amino acid sequences revealed six well supported lineages of avian astroviruses (Fig. 4), roughly corresponding to *Avastrovirus* species based on their genetic divergences. Also, we performed another phylogenetic analysis removing those sequences with possible recombination events (some members of GFAstV; Wood pigeon Astrovirus, WpiAstV; DAstV; CAstV; TAstV; and ANV), obtaining the same well supported clades, ruling out the possible influence that recombination could have had in our results.

The species *Avastrovirus 2* and *Avastrovirus 3* were recovered with maximum branch support, but *Avastrovirus* 1 formed a paraphyletic group with respect to *Avastrovirus* 3, which was included in its clade with the same uniqueness and statistical support as the two major clades of *Avastrovirus 1*. Therefore, if *Avastrovirus 1* and *Avastrovirus 3* are to be kept as different species, we propose distinguishing between two evolutionarily distinct groups within the species *Avastrovirus 1* (*Avastrovirus 1a* and *1b* in Fig. 4). The three sequences of Northern pintail named “Avastroviruses Group 3” by Chu *et al*.^17^ formed a distinct, early diverging clade, which we named *Avastrovirus 4*. The four newly discovered passerine astroviruses from French Guiana formed another distinct clade, which we provisionally named *Avastrovirus 5*. Between-group mean genetic distances for *Avastrovirus 4* (0.727–0.745) and *Avastrovirus 5* (0.672–0.745) are greater than genetic distances among the three previously described *Avastrovirus* species (0.550–0.680). The *Avastrovirus 5* clade had very long internal branches compared to other groups of the same phylogenetic level, and their members scored the highest intraspecific genetic distance in average (0.671).

## Discussion

Metagenomics has become a powerful tool, capable of characterizing the diversity of viral communities in different ecosystems. A large amount of novel astroviruses has been discovered during the last decade, which lead to the redefinition of the *Astroviridae* classification^12,13^. In this study, four novel and genetically distinct astroviruses, provisionally designated as PavAstV-1-4 have been described in cloacal samples from apparently healthy populations of Neotropical passerines from French Guiana.

Our results significantly expanded the known range of hosts of avastroviruses and they provide further insight into genetic diversity, evolution and population structure of these viruses. Earlier reports on astroviruses from passerines were scarce or absent, with only one representative member detected in a black-napped monarch (*Hypothymis azurea*) from Cambodia^27^. However, our findings put forward passerines as putative cornerstone hosts of these viruses in the avifauna of the interior primary forest of the Guianan shield. We documented the presence of astroviruses in 14 species of passerine birds of the families Thamnophilidae, Cardinalidae, Conopophagidae, Furnariidae, Tyraniidae and Turdidae. The avifauna of this region is largely isolated from bird migratory routes, with most species being local residents^28,29^. In fact, we have never captured Nearctic migrants in various years of fieldwork at the Nouragues reserve. Therefore, further research is needed to clarify whether passerines are important astrovirus hosts worldwide (which have remained undersampled in previous research), or the pattern we found is a local occurrence singularly evolved in a remote and isolated avifauna. In relation to this, it is important to note that we found higher community-level prevalence (4.9%) than the observed in other previous studies based on cloacal swabs (1.7% in a study conducted in Cambodia and Hong Kong^17^). The difference becomes still higher if it is restricted to passerine birds, for which only one individual tested positive out of 199 screened in Cambodia by Mendenhall *et al*.^27^. This represents 0.8% prevalence, comparable with 5.1% of passerines infected in French Guiana (20 positive out of 395 tested).

The four novel astroviruses found in French Guiana substantially contribute to broaden the genetic and phylogenetic diversity of the genus *Avastrovirus*. The phylogenetic analysis of ORF2 amino acid sequences of *Avastrovirus* in our study revealed wider phylogenetic diversity than previously thought for the respective genus. Our analysis added two new, early diverging groups to the known diversity of *Avastrovirus* genus, which before this study was composed of three recognized and accepted avastrovirus species (*Avastrovirus 1*, *2* and *3*)^30^. According to our phylogenetic analysis, *Avastrovirus 1* is a paraphyletic group. Based on phylogenetic evidence, *Avastrovirus* *3* should be a member of the same species, although the internal structure of *Avastrovirus 1* recommends dividing the whole group into three clades, with *Avastrovirus 1b* being sister to *Avastrovirus 3* and *Avastrovirus 1a* as a distinct, earliest diverging clade in that group. This topology, where *Avastrovirus 1* is not a monophyletic group, can be observed in previous phylogenetic analyses based on full-length ORF2 amino acid sequences^31–33^, although this matter is not addressed in these studies. The four sequences recovered from French Guianan passerines form a distinct novel clade (*Avastrovirus 5*) that is sister to the group formed by *Avastrovirus 1*, *2* and *3*, although its members are more divergent from one another than the members of other clades. The fact that passerine astroviruses from French Guiana cluster together but form a clade with long internal branches suggests the possibility that greater diversity of viruses exists in this region, hidden by incomplete sampling in our local study of birds from the Guianan shield. The diversity of *Avastrovirus* can be further broadened by the recognition of *Avastrovirus 4*, a group of early diverging viruses found in a relatively well-sampled group of birds (ducks). Astroviruses included in *Avastrovirus 4* already appeared forming a different clade when they were first described in both RdRp and ORF2 phylogenetic trees^17^. Our proposal of two new *Avastrovirus* species is supported by the large between-group mean genetic distances in the amino acid sequences of ORF2 introduced by these viruses, with higher values for *Avastrovirus 4* (astroviruses from Northern pintail) and *Avastrovirus* 5 (astroviruses from French Guianan passerines) than for the previously described species *Avastrovirus 1*, *Avastrovirus* 2 and *Avastrovirus* 3. Leaving aside the taxonomic debate, our results demonstrate that the real diversity of avian astroviruses is yet to be discovered, and sampling in remote areas can be critical to gain full insight into its distribution across regions and hosts.

The genomic organization of the novel passerine avastroviruses from French Guiana involved some differences on ORF layout. The genomes PasAstV-1 and PasAstV-3 have each ORF in a different reading frame, an especial characteristic that only occurs in the DAstV isolate described by Fu *et al*.^34^, since astroviruses have usually two ORFs on the same reading frame. PasAstV-4 has ORF1b in +1 frame, the same as ANV-1^24^. Furthermore, ORF1b and ORF2 from PasAstV-2 are on +1 reading frame, which is common in some *Mamastrovirus* and *Avastrovirus*. PasAstV-1-4 show the conserved tyrosine residue within the TEEEY-like domain in the VPg putative protein. The ORF sizes of astroviruses from French Guiana are more similar to those from *Mamastrovirus* than from *Avastrovirus*. The 5′ ends of the four passerine astroviruses are within *Astroviridae* range (11–85 nt)^35^, although they are longer than those described for other *Avastrovirus*. As in the European roller astrovirus isolate, phylogenetically related to *Mamastrovirus*, passerine astroviruses lack the gRNA promoter sequence on their 5′ end^36^. It is also interesting the *Mamastrovirus*-like structure present on the ribosomal frame-shift signal located between the ORF1a and 1b of PasAstV-1. Stop-codon location between AAAAAAC heptamer and the stem-loop in the ORF1a suggests that the ancestor of this astrovirus likely originated from a recombination event between *Mamastrovirus* and *Avastrovirus*, even though recombinations between the two genera have not been described so far^13^. Likewise, only one case of recombination in a human astrovirus between ORF1a and ORF1b has been described^37^. These *Mamastrovirus*-like features in avian astroviruses together with the wide genetic variety of *Astroviridae* have been proposed as a sign of possible cross-species transmission^36^.

The four astroviruses discovered in passerines of French Guiana, provisionally named as *Avastrovirus* 5, imply an increase in the diversity of *Astroviridae*, because although they have a typical *Avastrovirus* genomic structure, they also share some characteristics of the members of *Mamastrovirus*. Interestingly, only representatives of *Avastrovirus 5* have been found in birds from French Guiana (including a wide representation of avian lineages). One explanation for this could be that previously known diversity of astroviruses is mainly restricted to particular hosts (such as chickens, ducks or turkeys) which were not present in our study. Alternatively, French Guianan astroviruses likely represent an endemic diversity, which would persist in this remote and greatly isolated area as no migratory avian species or other major sources of interchange such as poultry trading occurs. Sampling typical hosts of *Astrovirus 1*, *2* and *3* in French Guiana would help answer this question. Astroviruses are present in the virome of some animals^19,38^, but there are no reports about astroviruses as a causative agent of disease in wild birds, so they could be only the hosts of these viruses. Therefore, our results pave the road for further research about the evolutionary origin, geographic distribution and ecological relationships of these viruses, an interest that can be extended to other virus families.

## Methods

### Sample collection

A random sampling of understory bird species was carried out in Pararé and Inselberg camps, in the Nouragues Natural Reserve, French Guiana (4°05′N, 52°40′W). The sampling area was located in a tropical rainforest, where average temperature is around 26 °C throughout the year and relative humidity is usually high. The climate is very wet in general, with annual precipitation exceeding 3,000 mm, although there is a dry season with considerably less rainfall between August and November. Birds were mist-netted in January 2016 (rainy season) and October-November 2016 (dry season). They were taken standard morphometric measurements, ringed to avoid repeated sampling of the same individuals, and released unharmed at the site of capture. Cloacal samples were collected using sterile swabs (Nerbe Plus), which were preserved in 800 µl of universal viral transport medium (VTM) (Becton Dickinson) and kept frozen until molecular analyses. A total of 406 cloacal samples from 72 bird species were collected.

### Sample processing and next generation sequencing

We selected 50 samples with abundant fecal matter in cloacal swabs, including as many different bird species as possible. We grouped them to create five pools of 10 samples each (hereafter pools 1–5). The samples were vortexed, and the swabs were squeezed to release epithelial cells. The VTM were centrifuged at 13,000 rpm for 1 min to pellet out epithelial cells. VTM were removed and the pellets resuspended in 250 µl of PBS. Then, samples were subjected to 2 freeze-thaw cycles at −80 °C to maximize the release of viral particles, and filtered through 0.45 µm pore-sized column filters at 8,000 rpm for 5 min to enrich viral particles in the flow-through. We took 50 µl of the filtrate of each sample and mixed them in five pools of 10 samples each. Each filtrate was treated with a mixture of nucleases (Turbo DNase, Ambion, Carlsbad, CA, USA; Baseline-ZERO, Epicenter, Madison, WI, USA; Benzonase, Novagen, San Diego, CA, USA; RNAse One, Promega, Fitchburg, WI, USA) to digest unprotected nucleic acids including host DNA/RNA. Finally, viral RNA/DNA was extracted with the MagMAX Viral RNA Isolation Kit (Thermo Fisher) according to the manufacturer’s instructions. Standard biosafety level-2 biocontainment measures were followed during the whole process. The extracted viral RNA and DNA were subjected after random RT-PCR amplification for library preparation by using QIAseq FX DNA Library Kit (Qiagen, Germany). Normalized samples were pooled and sequenced using 600-cycle (2 × 300 bp paired-end) MiSeq Reagent Kits v3 (Illumina, San Diego, CA) on a MiSeq platform. The generated raw reads were first qualitatively checked, with Phred quality score < 20 trimmed and filtered to remove polyclonal and low quality reads (< 55 bases long) using CLC workbench (Qiagen). The remaining filtered raw reads were de-novo assembled separately using Trinity v2.6.642^39^ and CLC workbench and compared with a non-redundant and viral proteome database (NCBI) using BLASTx with an E-value cut-off 0.001. The virus-like contigs and singlets were further compared to all protein sequences in non-redundant protein databases with a default E-value cutoff of 0.001. The viral metagenomics output has been visualized and analyzed in MEGAN^40^.

### Prevalence of astroviruses in the cloacal swabs

A total of 356 cloacal samples, which were not subjected to deep-sequencing, were processed differently. Only half of the VTM volume (400 µl) was taken after vortexing. Swabs were squeezed and discarded, samples were centrifuged and VTM was removed in the same way as for samples analyzed by deep sequencing. However, the pellets were resuspended in 40 µl of PBS, avoiding freeze-thaw cycles, filtering or nuclease treatment. These samples were grouped in 36 pools, 34 of which contained 10 individuals, one of 11 individuals and one of five individuals. Viral RNA/DNA was extracted using the same protocol as for samples subjected to deep sequencing.

Degenerate primers described by Todd *et al*.^23^ for pan-astrovirus RT-PCR, which amplify a fragment of the RNA-dependent RNA polymerase (RdRp) region of the ORF1b, were used to detect the presence of astroviruses. Due to the presence of PCR inhibitors in feces, 5 μl of a 10^−1^ dilution from the individual RNA/DNA extraction were added as a template. PCR assays were performed with a final volume of 25 μl reaction mixture consisting of 5 μl extracted RNA/DNA, 12.5 µl *Verso 1-Step RT-PCR Hot-Start Kit* (Thermo Fisher), 0.5 µl of each primer (5 pmol), and ddH_2_O up to 25 µl using a cycling condition as follows: synthesis of cDNA at 50 °C for 15 min followed by denaturation at 95 °C for 15 min; 45 amplification cycles were performed at 95 °C for 20 s, 45 °C for 30 s, 72 °C for 1 minute. Final extension was at 72 °C for 5 min.

### Genomic analysis of the four novel PasAstV

The ORFs were predicted using ORFfinder (NCBI: https://www.ncbi.nlm.nih.gov/orffinder/). TMHMM server v2.0^41^ was employed for prediction of transmembrane helices in proteins; cNLS Mapper^42^ for nuclear localization signals and NCBI Conserved domains (NCBI: https://www.ncbi.nlm.nih.gov/Structure/cdd/wrpsb.cgi) for the location of serine protease domains, RdRp and the conserved region of the capsid. Secondary structures were predicted by FoldIndex^43^ for locating the viral protein associated with the genome (VPg), and by Multicoil scoring form^44^ for locating the coiled-coil regions. Hairpin loops were analysed using Mfold^45^.

### Phylogenetic and taxonomic analysis

Genome sequence analysis, genomic organization and multiple alignments were performed using Geneious v11 (Biomatters, New Zealand), EditSeq and SeqMan tools of the DNASTAR 5.0 software package (DNASTAR, Madison, WI), and BioEdit Sequence Alignment Editor^46^.

Evolutionary relationships of the four novel PasAstV with representative avastroviruses were determined by the construction of the phylogenetic tree based on amino acid sequences of the ORF2 gene. The initial complete ORF2 nucleotide data set was pruned from the potential recombinants using the various methods for recombination detection implemented in RDP4^47^. Recombinations were considered only when they were detected by more than three out of seven methods having significant p-values (p < 0.05). The phylogenetic tree based on ORF2 amino acid sequences was inferred using the Bayesian Markov chain Monte Carlo (MCMC) approach available in BEAST v1.84^31^. The analysis was performed under the best fit amino acid substitution model identified as LG + Г + I + F using Bayesian Information Criterion implemented as the model selection framework in jModelTest^48^ and MEGA X^49^. Monte Carlo Markov Chains (MCMC) were run for 10^7^ generations, sampling every 1,000 trees. Traces were inspected for convergence with Tracer 1.5^50^. The 10,000 resulting trees were summarized with TreeAnnotator v2.1.2^50^ and the phylogeny with branch posterior probabilities was displayed in FigTree v1.4.2 (http://tree.bio.ed.ac.uk/software/figtree/). We also computed pairwise amino acid genetic distances (p-dist) of the capsid region (ORF2) between isolates, between astrovirus groups and between members of the same group. To do so, we used MEGA X program using the bootstrap method with 1000 replicates and partial deletion (95%). The identity matrices were generated with the MUSCLE tool^51^ (https://www.ebi.ac.uk/Tools/msa/muscle/), and transformed to HeatMaps with Morpheus software (https://software.broadinstitute.org/morpheus).

### Ethics statement

All methods were carried out in accordance with European Union and national French regulations. Capture, sampling and transport of samples were authorised by the Service of Natural Environments, Biodiversity, Sites and Landscapes, Regional Directorate for the Environment, Planning and Housing at French Guiana (license 030418). The experimental protocols were approved by the Committee on Animal Testing of Complutense University (CEA-UCM, authorisation number 44-2016).

## Supplementary information

Table S1
