# Supplementary material for: A novel group of avian astroviruses from Neotropical passerine birds broaden the diversity and host range of Astroviridae
Source: Sci Rep. 2019 Jul 2;9:9513. doi: 10.1038/s41598-019-45889-3 (PMC6606752; doi:10.1038/s41598-019-45889-3)
Supplement: Supplementary file 1 — Table S1 [file 41598_2019_45889_MOESM1_ESM.docx]

**A novel group of avian astroviruses from Neotropical passerine birds broaden the diversity and host range of *Astroviridae***

**Authors:** Izaskun Fernández-Correa^1^, Daniel A. Truchado^1,2^, Esperanza Gomez-Lucia^3^, Ana Doménech^3^, Javier Pérez-Tris^2^, Jonas Schmidt-Chanasit^4,5^, Daniel Cadar^4^ and Laura Benítez^1^

**Supplementary material**

**Table S1.** Comparison of the conserved domains in the protein sequences of the four novel PasAstV. The number of conserved domains in each genome is underlined. The size in amino acids of each sequence is shown (in parentheses). NLS: nuclear localization signal; VPg: viral genome-binding protein; RdRp: RNA-dependent RNA polymerase.

| **ORF1a (aa)** | **PasAstV-1 (908)** | **PasAstV-2 (902)** | **PasAstV-3 (881)** | **PasAstV-4 (926)** |
| --- | --- | --- | --- | --- |
| **Transmembrane helix** | 5: 161-183, 328-350,  362-384,  399-416,  423-445 | 6: 168-190, 308-330, 340-362, 369-388, 398-420, 427-449 | 5: 298-320, 330-352, 359-378, 388-410, 417-439 | 4:183-202, 342-364, 374-396, 443-465 |
| **NLS** | 2: 154 and 840 | 1: 822 | 1: 843 | 0 |
| **Coiled-coils** | 1: 126-162 | 1: 703-721 | 1: 127-155 | 1:138-158 |
| **Serinprotease** | 1: 507-630 | 1: 517-627 | 1: 491-616 | 1:518-644 |
| **VPg** | 1: 760-820 | 1: 720-301 | 1: 706-880 | 1:741-926 |
| **ORF1b (aa)** | **PasAstV-1 (526)** | **PasAstV-2 (480)** | **PasAstV-3 (490)** | **PasAstV-4 (402)** |
| **RdRp** | 127-398 | 131-410 | 142-419 | 57-334 |
| **ORF2 (aa)** | **PasAstV-1 (755)** | **PasAstV-2 (767)** | **PasAstV-3 (752)** | **PasAstV-4 (788)** |
| **Conserved** | 36-396 | 83-406 | 68-408 | 84-459 |
| **Hypervariable** | 397-755 | 407-767 | 408-752 | 459-788 |
